# Behavioural and neurodevelopmental characteristics of SYNGAP1

**DOI:** 10.1186/s11689-024-09563-8

**Published:** 2024-08-15

**Authors:** Nadja Bednarczuk, Harriet Housby, Irene O. Lee, IMAGINE Consortium, David Skuse, Jeanne Wolstencroft

**Affiliations:** Behavioural and Brain Sciences Unit, Population, Policy and Practice Department, University College London (UCL) Great Ormond Street Institute for Child Health, 30 Guilford Street, London, WC1N 1EH UK

**Keywords:** SYNGAP1, Intellectual Disability, Neurodevelopment, Behaviour, Autism

## Abstract

**Background:**

SYNGAP1 variants are associated with varying degrees of intellectual disability (ID), developmental delay (DD), epilepsy, autism, and behavioural difficulties. These features may also be observed in other monogenic conditions. There is a need to systematically compare the characteristics of SYNGAP1 with other monogenic causes of ID and DD to identify features unique to the SYNAGP1 phenotype. We aimed to contrast the neurodevelopmental and behavioural phenotype of children with SYNGAP1-related ID (SYNGAP1-ID) to children with other monogenic conditions and a matched degree of ID.

**Methods:**

Participants were identified from the IMAGINE-ID study, a UK-based, national cohort study of neuropsychiatric risk in children with ID of known genetic origin. Thirteen children with SYNGAP1 variants (age 4–16 years; 85% female) were matched (2:1) with 26 controls with other monogenic causes of ID for chronological and mental age, sex, socio-economic deprivation, adaptive behaviour, and physical health difficulties. Caregivers completed the Development and Wellbeing Assessment (DAWBA) and physical health questionnaires.

**Results:**

Our results demonstrate that seizures affected children with SYNGAP1-ID (84.6%) more frequently than the ID-comparison group (7.6%; *p* =  < 0.001). Fine-motor development was disproportionally impaired in SYNGAP1-ID, with 92.3% of children experiencing difficulties compared to 50% of ID-comparisons(*p *= 0.03). Gross motor and social development did not differ between the two groups. Children with SYNGAP1-ID were more likely to be non-verbal (61.5%) than ID-comparisons (23.1%; *p* = 0.01). Those children able to speak, spoke their first words at the same age as the ID-comparison group (mean = 3.25 years), yet achieved lower language competency (*p* = 0.04). Children with SYNGAP1-ID compared to the ID-comparison group were not more likely to meet criteria for autism (SYNGAP1-ID = 46.2%; ID-comparison = 30.7%; *p* = .35), attention-deficit hyperactivity disorder (15.4%;15.4%; *p* = 1), generalised anxiety (7.7%;15.4%; *p* = .49) or oppositional defiant disorder (7.7%;0%; *p* = .15).

**Conclusion:**

For the first time, we demonstrate that SYNGAP1-ID is associated with fine motor and language difficulties beyond those experienced by children with other genetic causes of DD and ID. Targeted occupational and speech and language therapies should be incorporated early into SYNGAP1-ID management.

**Supplementary Information:**

The online version contains supplementary material available at 10.1186/s11689-024-09563-8.

## Introduction

The SYNGAP1 gene is one of the more common genetic causes of intellectual disability (ID), with an estimated prevalence of 0.5–1% of children with ID [1]. SYNGAP1 encodes a Ras-specific GTPase-activating protein, SynGAP, which is localised to the post-synaptic density of cortical neurons and influences important cellular signalling pathways in growth and survival [2, 3]. It plays a complex role in neurodevelopment and ongoing neurological function [3]. For instance, SynGAP regulates synaptic formation, maturation and plasticity in critical periods of cortical development [3–5]. Non-synaptic functions have also been linked to SynGAP, including axonal outgrowth and neuronal migration [5, 6]. Any disruption in typical SynGAP function, can result in abnormal cortical connectivity and disrupted neuronal signalling, which can in turn impair cognitive function [3, 5]. Indeed, SynGAP has been shown to be particularly important for learning and memory [3, 7]. Consequently, rare coding variants in SYNGAP1, which encode SynGAP, are strongly associated with intellectual disability (ID) and developmental delay[1]. Children with SYNGAP1-related ID (SYNGAP1-ID) may also experience seizures, hypotonia, digestive and sleeping difficulties [8].

The developmental phenotype associated with SYNGAP1-ID has only been described in case series. Most children have global developmental delay, but the severity of the impairment varies [8, 9]. A study of the neurodevelopmental profile of 17 children with SYNGAP1 reported a mean age of walking of over 2 years and most children speaking their first words at approximately 2.5 years [10]. A significant proportion of children also remain non-verbal [11]. It is unclear which features, if any, of the developmental phenotype differ from that observed in other genetic disorders causing developmental delay.

Various neurodevelopmental conditions have been described in SYNGAP1-ID, in particular elevated rates of autism [9, 12–14]. In the largest cohort of 57 individuals, an autism rate of 53% was reported [9]. Some case series have mirrored these autism rates, whilst others report rates of up to 73% [15, 16]. The SYNGAP1 gene has been considered to have high degree of autism specificity [17, 18]. Aggressive behaviour affects up to 60% of children [16]. Caregivers have reported varying degrees of impulsivity and self-injurious behaviour [11, 16]. Sensory processing impairments are common among children with SYNGAP1-ID [11]. Rates of ADHD, conversely, appear to be low, affecting only 7% of individuals with SYNGAP1-ID [15].

Few studies have compared the behavioural phenotype of SYNGAP1-ID to that of other monogenic causes of ID. Two recent research studies have attempted to delineate the SYNGAP1-ID phenotype by comparing the behavioural profile of those with SYNGAP1-ID to those with other disorders affecting synaptic dysfunction, specifically Phelan-McDermid Syndrome. Naveed et al*.* compared Social Responsiveness Scale scores [19] in the two conditions, finding similar levels of difficulty in social interaction [20]. Lyons-Warren et al*.* utilised the Short Sensory-Profile 2 [21] to assess sensory processing. Atypical sensory processing was observed in both SYNGAP1-ID and Phelan-McDermid Syndrome [22]. Beyond these studies, no other study has employed standardised developmental and behavioural measures to compare SYNGAP1-ID to other genetic causes of ID.

We aimed to systematically assess the behavioural and neurodevelopmental phenotype of SYNGAP1- ID and compare this to a matched comparison group with an equivalent level of intellectual disability caused by other, heterogenous monogenic disorders. We sought to identify behavioural and neurodevelopmental patterns that are unique to children with SYNGAP-ID.

## Methods

### Participants

Participants with SYNGAP1 variants were identified from the IMAGINE-ID (the Intellectual Disability and Mental Health: Assessing the Genomic Impact on Neurodevelopment) study. IMAGINE-ID is a large, national cohort study of children with intellectual disability or developmental delay of known genetic origin. Children were recruited to IMAGINE via regional genetic services, charities, support groups and by self-referral [23]. Molecular genetic diagnoses had to be established by an National Health Service (NHS) accredited diagnostic laboratory and all pathogenic variants were categorised according to the American College of Medical Genetics and genomics guidelines [24]. Participants with pathogenic or likely pathogenic genetic variants were included. Parents or guardians provided consent on behalf of children younger than 16 years and for those older than 16 years lacking capacity, consultees acted on their behalf. IMAGINE-ID was approved by the London Queen Square Research Ethics committee (14/LO/1069).

A comparison group with intellectual disability due to other genetic variants were also identified from the IMAGINE-ID cohort. Participants in the comparison group were selected using a matched block design. Blocks were matched for age, sex, level of socio-economic deprivation, physical health difficulty, and degree of developmental delay. The degree of developmental delay was assessed using caregiver-reported mental age[25] and the Adaptive Behaviour Assessment 3rd Edition (ABAS-3) generalised adaptive composite score [26]. Within blocks, participants were selected at random at a 2:1 ratio. Genetic variant did not affect ID-comparison group participant inclusion. The genetic variants present within the ID-comparison group are displayed in Table 1. Full details of genetic variants for the SYNGAP1-ID and ID-comparison group are listed in Supplementary Table 1 and 2 respectively.

**Table 1:** Genetic variants in ID-comparison group

ASXL3	KCNQ2
ANKRD11	KDM6A
BBS10	GRIN2B
CNOT3	NF1
CREBBP	NSD1
CTNNB1	PPM1D
DDX3X	SPRED1
DONSON	TRIO
EP300	UBE3A
FMR1	WAC

List of genetic variants present within the ID-comparison group.

### Behavioural and developmental phenotype

Behavioural and developmental data were collected via online questionnaires completed by caregivers on behalf of their child. This included the Developmental and Wellbeing assessment (DAWBA) and the Strengths and Difficulties Questionnaire (SDQ). The DAWBA is a structured psychiatric interview which assesses developmental and behavioural difficulties alongside neuropsychiatric diagnoses [27]. Provided information was reviewed and rated according to DSM-5 diagnostic criteria by two experienced clinicians. The presence of autism, attention-deficit hyperactivity disorder (ADHD), generalised anxiety disorder, oppositional defiant disorder and conduct disorder were assessed. Inter-rater reliability has been reported in previous IMAGINE-ID publications [23, 28]. The SDQ is a parent-rated questionnaire that measures emotional symptoms, conduct problems, hyperactivity/inattention, peer relationship problems and prosocial behaviour. A total difficulty score is calculated from the first four sub-scales. Higher scores reflect greater emotional and behavioural difficulties [29].

Adaptive functioning was assessed with the Adaptive Behaviour Assessment (ABAS-3) and caregiver-reported mental age [26]. Language competency was assessed by dividing caregiver estimated language age by the child’s chronological age, with values of one indicating that the child’s language age equalled their chronological age. Caregivers also completed a physical health questionnaire to assess any relevant past medical history.

### Statistical analysis

Statistical analysis was performed in R (Version 4.3.0). All data were tested for normality using Shapiro-Wilks tests. Normally distributed data were compared using t-tests. Non-normally distributed data were assessed using non-parametric tests, including Mann–Whitney-U testing. Bonferroni corrections were applied throughout to account for multiple testing. Wilcox rank effect sizes (r) were calculated for significant results. Categorical data were tested using chi-squared tests with Yates’s correction to account for sample size.

## Results

### Participant demographics

Thirteen children with SYNGAP1 variants were identified from the IMAGINE-ID cohort alongside 26 children in the ID-Comparison group (Table 2). Adaptive functioning was extremely low, that is 3 standard deviations below the population mean in the SYNGAP1 group (median 57.7; range 46–75), which was matched for in the ID-comparison group (median 51.1; range 46–71; *p* = 0.19).

**Table 2:** Participant Demographics

*Median Score (range)*	**SYNGAP1- ID (*n* = 13)**	**ID-Comparison Group (*n* = 26)**	***p***
**Age**	Mean: 8.8 years (range: 4–16)	Mean: 9.4 years (range: 4–16)	0.63
**Sex**	Female: *n* = 11; Male *n* = 2	Female: *n* = 23; Male: *n* = 3	1.0
**IMD Decile**	8 (2–10)	8 (2–10)	0.31
**Mental Age**	3.62 years (1–10 years)	4.32 years (0–8 years)	0.37
**ABAS Generalised Adaptability Composite**	57.7 (46–75)	51.1 (46–71)	0.19
**Physical Health**	Very good: *n* = 1	Very good: *n* = 2	0.18
	Good: *n* = 2	Good: *n* = 13	
	Fair: *n* = 8	Fair: *n* = 8	
	Bad: *n* = 1	Bad: *n* = 3	
**Physical Health Co-morbidities**	Seizures *n* = 11	Seizures *n* = 2	< 0.001
	Movement difficulties *n* = 10	Movement difficulties *n* = 17	0.48
	Respiratory co-morbidities *n* = 1	Respiratory co-morbidities *n* = 12	0.06
	Cardiac co-morbidities *n* = 1	Cardiac co-morbidities *n* = 4	0.48
	Gastrointestinal co-morbidities *n* = 6	Gastrointestinal co-morbidities *n* = 12	0.79
	Musculoskeletal co-morbidities *n* = 1	Musculoskeletal co-morbidities *n* = 5	0.52
**Neuropsychiatric Co-morbidities**	Autism-spectrum disorder *n* = 6	Autism-spectrum disorder *n* = 8	0.55
	ADHD *n* = 2	ADHD *n* = 4	1.0
	Generalised Anxiety *n* = 1	Generalised Anxiety *n* = 4	0.49
	Oppositional defiant disorder *n* = 1	Oppositional defiant disorder *n* = 0	0.15
**Mutation Type**	Frameshift *n* = 6	Frameshift *n* = 10	
	Stop gained *n* = 4	Missense *n* = 6	
	Splice site *n* = 2	Stop gained *n* = 7	
	Inframe deletion *n* = 1	Splice site *n* = 1 Expansion *n* = 1 Unknown *n* = 1	

Table summarising and comparing the demographic details, adaptive functioning, and physical health data for the SYNGAP1-ID cohort and the ID comparison group. Statistical significance is highlighted with *p*-values.

Children with SYNGAP1-ID were born at a mean of 38 weeks (range 34–42 weeks) with a mean birthweight of 3.0 kg (2.3kg -3.6kg). Children in the ID-comparison group were born at a mean of 38.8 weeks (range: 33–42 weeks; *p* = 0.51) with a birth weight of 3.2 kg (1.9 kg – 4.5 kg; *p* = 0.769). Physical health co-morbidities are shown in Table 2. Both SYNGAP1-ID and the ID-comparison group experienced gastrointestinal difficulties (SYNGAP1-ID *n* = 6; ID-comparator *n* = 12; *p* = 0.79, of which constipation and gastro-oesophageal reflux were most common. Respiratory co-morbidities were more common, although not significantly, in the ID-comparison group (*n* = 12; 8 = recurrent chest infections, 4 = asthma) compared to SYNGAP1-ID (*n* = 1; recurrent chest infection, *p* = 0.06). Rates of cardiac and musculoskeletal difficulties were similar in both groups [Table 2].

### Neurological Symptoms & Epilepsy

The most common neurological symptoms experienced by children with SYNGAP1-ID were seizures. Seizures affected children with SYNGAP1-ID (*n* = 11; 84.6%) more frequently than the ID-comparison group (*n* = 2; 7.6%, *p* =  < 0.001), most commonly absence seizures (*n* = 8; 72.7%) and atonic seizures (*n* = 3; 23.1%). No neonatal seizures were reported for children with SYNGAP1 ID or the comparison group. Of those children with SYNGAP-ID experiencing seizures, ten (90.9%) were receiving anti-epileptic medication.

Muscle and movement difficulties were also frequently reported in children with SYNGAP1-ID (*n* = 10; 76.9%). Nine children (90%) experienced hypotonia, three (30%) experienced ataxia, two (20%) experienced hypertonia. The ID comparison group experienced similar rates of muscle and movement difficulties (*n* = 17; 65.4%- *p* = 0.483). Ataxia (ID-comparison *n* = 1; 3.8%; SYNGAP1-ID = 3; 23.1%; *p* = 0.09) displayed a trend towards being more common in children with SYNGAP1-ID. Rates of hypotonia in the ID-comparison group (*n* = 14; 82.4%-*p* = 0.58) were not significantly different from SYNGAP1-ID. No cases of visual or hearing impairment were reported in children with SYNGAP1-ID.

### Developmental milestones

Three children with SYNGAP1-ID (23%) experienced developmental regression compared to only one child (3.8%) in the ID comparison group (*p* = 0.14).

#### Gross & fine motor

Most children with SYNGAP1-ID (*n* = 12; 92.3%) and the ID-comparison group (*n* = 24; 92.3%) were able to sit and walk independently. There was no difference in the age of walking observed between SYNGAP1-ID (mean 2.3 years) and the comparison group (mean 2.23 years; *p* = 0.30; Wilcox effect size [r] = 0.17).

Fine motor development, however, was significantly affected in children with SYNGAP1-ID. Only one child with SYNGAP1-ID was able to independently do up buttons and achieved this milestone at 8 years of age. Contrastingly, 50% (*n* = 13) of the ID-comparison group were able to do-up buttons (*p* = 0.03) and achieved this milestone at a mean of 7.6 years.

#### Social

Social development was assessed using age of smiling. There was no significant difference in the number of children able to smile by two months (upper limit of normal range) between SYNGAP1-ID (*n* = 7; 53.8%) and ID-comparisons (*n* = 7; 26.9%; *p* = 0.13). Among the other children, the mean age of smiling in SYNGAP1-ID was 7 months (range 3–15 months) and 4.5 months (range 3–7 months) for the ID-comparison group, with no significant difference in achieving this milestone across groups (*p* = 0.61; r = 0.15).

#### Speech & language

**Fig. 1:**
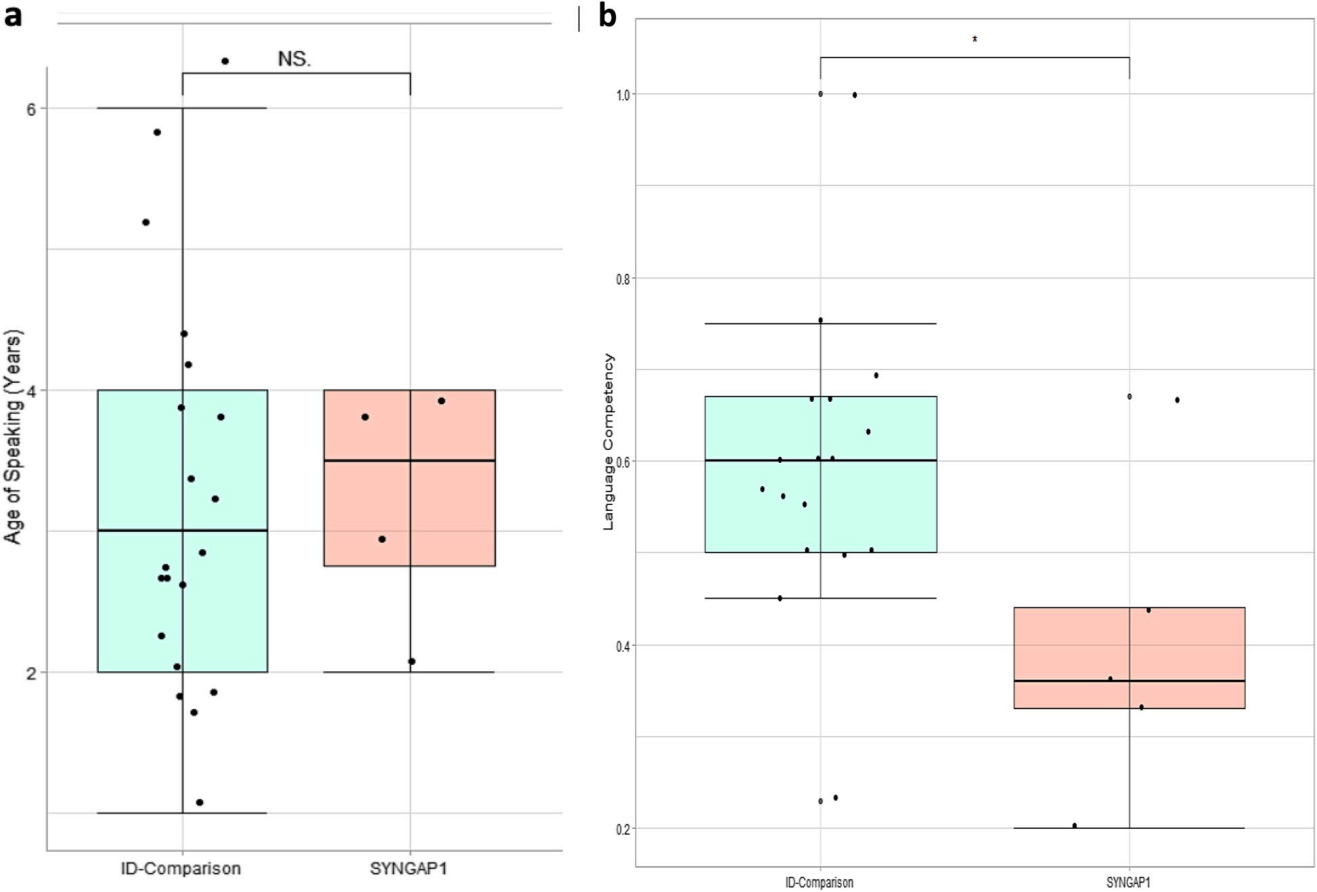
**a** Box plot demonstrating the age of speaking their first words for children with SYNGAP1-ID and the ID-comparison group. **b** Box plots highlighting spectrum of language competency within children able to speak in the SYNGAP1-ID and ID-comparison group. Even when matched for developmental level, language competency in SYNGAP1-ID is lower than in the ID-comparison group

Children with SYNGAP1-ID were more likely to be non-verbal (*n* = 8; 61.5%) than the ID-comparison group (*n* = 6;23.1%; *p* = 0.01). SYNGAP1-ID children that were able to speak (*n* = 5, mean = 3.3 years) achieved this milestone at the same age as the comparison group (mean = 3.3 years; *p* = 0.84)[Fig. 1a]. Among children able to speak, caregiver-estimated language competence (i.e., the child’s language age divided by their chronological age) was moderately lower in SYNGAP1-ID (mean = 0.4) compared to the ID-comparison group (mean = 0.6; *p* = 0.040, Wilcox effect size *r* = 0.44) [Fig. 1b], with language competency values of 1 indicating that the child’s language age is equal to their chronological age.

### Neuropsychiatric diagnoses & behavioural difficulty

#### Emotional and behavioural adjustment

There was no significant difference in the total SDQ score between SYNGAP1-ID (median 20; range 8–27) and the ID-comparison group (median 18.5; 11–31; *p* = 0.98). Most children with SYNGAP1-ID (53.8%) scored in the ‘very high’ severity band of the DAWBA, indicative of difficulties experienced by the extreme 10% of the population [29]. This was higher, although not significantly, than the ID-comparison group (*n* = 10, 38.5%; *p* = 0.67; *r* = 0.005). SYNGAP1-ID Total SDQ scores did not significantly differ between non-verbal children (*n* = 17; 8–31) and those able to speak (*n* = 19; 10–30; *p* = 0.35). There were no significant differences in SDQ sub-scales between groups [Table 3].

**Table 3:** SDQ Sub-scales

*Median Score (range)*	**SYNGAP1-ID**	**ID- Comparison**	**p-value**
Emotional Symptoms	5 (0–9)	4.5 (0–10)	0.98
Conduct Problems	3 (0–5)	2 (0–7)	0.59
Hyperactivity/Inattention	8 (3–10)	8 (3–10)	0.93
Peer Relationship Problems	4 (1–9)	4 (0–9)	0.74
Total Difficulty Score *(Severity band)*	20 (8–27) *Very high*	18.5 (11–31) *High*	0.98

Comparison of SDQ total difficulty score and sub-scale scores between SYNGAP1-ID and ID-comparison group. Statistical significance is shown in *p*-value column.

#### Psychiatric Diagnoses

The incidence of DSM-5 autism diagnosis among children with SYNGAP1-ID (*n* = 6; 46.2%) was greater, although not significantly, compared to the ID-comparison group (*n* = 8; 30.7%; *p* = 0.55). 7.7% (*n* = 1) of children with SYNGAP1-ID met the diagnostic criteria for generalised anxiety compared to 15.3% (*n* = 4) of the ID-comparison group (*p* = 0.49). Rates of ADHD (SYNGAP1-ID *n* = 2; 15.3%; ID-comparison n = 4; 15.3%; *p* = 1) and oppositional defiant disorder (SYNGAP1-ID *n* = 1; 7.7%; ID-comparison *n* = 0; *p* = 0.15) were not different across groups.

We also interrogated other behavioural features previously reported in the literature. Temper outbursts occurred in 12 (92.3%) of SYNGAP1-ID children and 25 (96.2%) of the ID-comparison group (*p* = 0.46). In six SYNGAP1-ID children (50%), temper outbursts involved aggressive behaviour, which did not differ from the trend observed in the comparison group (*n* = 14; *p* = 0.26). Self-injurious behaviour, such as head-banging and skin-picking, was present in 2 children (15.4%) with SYNGAP1-ID and 8 children (30.7%) from the ID-comparison group (*p* = 0.16). Children with SYNGAP1-ID more frequently displayed fascination with particular sensations (*n* = 9; 69.3%) compared to the ID-comparison group (*n* = 10; 38.5%- *p* = 0.17). Hyposensitivity to pain was observed in both children with SYNGAP1-ID (*n* = 11; 84.6%) and ID-comparisons (*n* = 20; 76.9%; *p* = 0.92).

## Discussion

We report the first systematic comparison of the neurodevelopmental and behavioural phenotype of SYNGAP1-ID to children with the same level of intellectual disability due to other heterogeneous genomic conditions. Our results highlight a specific pattern of neuro-behavioural characteristics that should be a focus for clinical care. These characteristics include significant global developmental delay, particularly impacting fine motor and speech and language development, gait abnormalities, autism, and in particular epilepsy. There was a striking propensity for children with SYNGAP1-ID to experience seizures, of which absence and atonic seizures were most commonly observed. Our findings on seizures support the existing literature on the seizure phenotype in SYNGAP1 [9].

Another unique feature of the SYNGAP1 developmental profile is the pronounced motor control difficulties. Although most children were able to walk independently, ataxia occurred more frequently in SYNGAP1-ID. Hypotonia, however, was a common feature among both groups. This mirrors previous reports of gait abnormalities, particularly ataxia, in this cohort [9, 15]. Locomotor abnormalities have been replicated in SYNGAP1 animal models, which may mirror observed gait abnormalities [30]. Fine motor skills were especially affected in SYNGAP1-ID, with only one child able to do up buttons compared to 50% of the ID-comparison group. Previous case series have reported some delays in fine motor ability, but have not considered functional outcomes [15].

Speech development is also disproportionally affected in SYNGAP1-ID, beyond delays observed in children matched for degree of developmental delay. SYNGAP1-ID children are not only more likely to be non-verbal, but also achieve lower levels of language competency. Similar findings were described in a recent case series, which also highlighted limited language attainment for those children able to speak [15]. Interestingly, we did not observe a difference in emotional and behavioural difficulties, as assessed by SDQ scores, between non-verbal children and those able to speak. Difficulties in language development may be explained by sensory processing difficulties [22]. SYNGAP1 mutations have been shown to lead to dysregulated cortical sensory system development, including atypical sensory map organisation [3, 31]. This results in distorted sensory processing, including the processing of incoming auditory signals. Altered electrophysiological responses to auditory stimuli have been demonstrated in individuals with SYNGAP1 variants when compared to individuals with Trisomy 21 and neurotypical controls [32]. Impaired perception and processing of auditory signals may adversely affect speech and language development, which may contribute to the language delays we observed in our cohort. As such, speech and language therapy should be a therapeutic priority.

The above difficulties, including locomotor, spatial learning, and sensory processing abnormalities, may be further explained by multiple down-stream effects of atypical synaptic formation and function caused by SYNGAP1 mutations [3]. The mutations result in premature functional maturation of excitatory neurones [33]. This in turn results in abnormal cortical circuits and connectivity, which may explain SYNGAP1’s impact on cognition. It may also disrupt more specific processes required to develop fine motor control and language skills [3, 33]. Similarly, atypical cortical connectivity may contribute to the previously described abnormal development of the brain’s sensory systems [3, 33]. Aberrant synaptic plasticity during critical periods of development can also inhibit activity-dependent synapse formation and strengthening, which in turn hinders learning [5, 7, 34]. Lastly, abnormal maturation of excitatory neurones and synapses can result in an imbalance between excitatory and inhibitory neuronal connections (E/I imbalance; Clement et al., [35]). E/I imbalance has been proposed as a hypothesis underlying the development of autism [36] and may explain the high rate (46% of SYNGAP1-ID children) of autism observed in our cohort, which is consistent with that of previous reports [9, 15].

The strength of this study is that it is the first systematic exploration of the neurodevelopmental and behavioural differences between SYNGAP1-ID and children with ID of genetic origin and an equivalent level of developmental delay and ID. Hereby, we identify behavioural and developmental features unique to SYNGAP1-ID. Assessing children presenting with neurodevelopmental delay or ID due to a suspected genetic diagnosis is often challenging due to significant overlap in phenotypic features between conditions. By comparing SYNGAP1-ID to a heterogenous group of monogenic conditions all associated with ID we aimed to mimic this clinical challenge and further aid clinicians in identifying SYNGAP1-associated features and providing appropriate counselling of children and their families. However, the heterogeneity within our comparison group limits our ability to draw conclusions about the impact of genetic function and mutation type on the observed phenotypic variation. We must also acknowledge that our sample size may limit the power of our study to detect significant differences in certain characteristics, such as autism, sensory sensitivities, and motor difficulties. Future work should focus on large, pooled cohorts to confirm our findings and further bridge the gap between genotypic diagnosis and phenotypic presentations in SYNGAP1-ID. There is also a need to assess the longitudinal development of these children and consider the clinical utility of therapy implementation.

## Conclusion

In conclusion, our results demonstrate that children with SYNGAP1-ID are more prone to seizures and motor difficulties, such as ataxia, than children with other monogenic conditions leading to ID. They also experience greater difficulties in fine motor and speech and language development as well as higher rates of autism when compared to children with equal levels of intellectual disability due to other, heterogenous genomic conditions. Identification of features specific to the SYNGAP1 phenotype can not only help guide diagnosis and clinical counselling, but also provide clinically relevant endpoints for future therapeutic trials. This will be particularly relevant in the promising advancement of animal-models of gene re-activation therapeutic approaches, which can lead to improvements in seizure threshold, learning, memory, and cognitive function [37]. Our findings also provide strong evidence for the implementation of more targeted therapeutic interventions in children with SYNGAP1, such as early speech and language and occupational therapy support.

### Supplementary Information

Supplementary Material 1.

## Data Availability

The full phenotypic IMAGINE dataset is available from the UK Data Archive under special licence access (SN 8621). Requests for genotype or linked genotypic-phenotypic data can be made through the IMAGINE data access committee.

## Abbreviations

ID: Intellectual Disability
DD: Developmental delay
SYNGAP1-ID: SYNGAP1-related intellectual disability
ADHD: Attention deficit hyperactivity disorder
DAWBA: Developmental and Well-being Assessment
ABAS: Adaptive Behaviour Assessment System
SDQ: Strengths and Difficulties Questionnaire
E/I imbalance: Excitatory and inhibitory neuronal imbalance
