# Supplementary material for: Behavioural and neurodevelopmental characteristics of SYNGAP1
Source: J Neurodev Disord. 2024 Aug 15;16:46. doi: 10.1186/s11689-024-09563-8 (PMC11325819; doi:10.1186/s11689-024-09563-8)
Supplement: Supplementary file 1 — Supplementary Material 1. [file 11689_2024_9563_MOESM1_ESM.docx]

**Supplementary Material:**

**Table 1: SYNGAP1 Genetic Variants**

| **Gene** | **Mutation type** | **Variant Type** | **Inheritance** | **Ref Sequence** | **Variant** |
| --- | --- | --- | --- | --- | --- |
| SYNGAP1 | Frameshift | SNV | De novo | NM_006772 | c.1347_1348A>TfsX4 |
| SYNGAP1 | Frameshift | SNV | De novo | NM_006772 | c.1167_1168G>QfsX26 |
| SYNGAP1 | Splice site | SNV | De novo | NM_006772 | - |
| SYNGAP1 | Stop gained | SNV | De novo | NM_006772 | c.1922S>X |
| SYNGAP1 | Stop gained | SNV | Maternal | NM_006772 | c.3244Q>X |
| SYNGAP1 | Splice site | SNV | De novo | NM_006772 | - |
| SYNGAP1 | Inframe deletion | SNV | Unknown | NM_006772 | c.2829_2846G_Gdel |
| SYNGAP1 | Stop gained | SNV | Unknown | NM_006772.2 | c.322K>X |
| SYNGAP1 | Stop gained | SNV | De novo | NM_006772 | c.638K>X |
| SYNGAP1 | Frameshift | SNV | De novo | NM_006772 | c.365A>CfsX42 |
| SYNGAP1 | Frameshift | SNV | Unknown | NM_006772 | c.3477N>GfsX8 |
| SYNGAP1 | Frameshift | SNV | De novo | NM_006772 | c.2621_2655S>RfsX4 |
| SYNGAP1 | Frameshift | SNV | Unknown | NM_006772 | c.1435R>PfsX14 |
| *SNV=* Single nucleotide variant | | | | | |

**Table 2: ID-Comparison Group Genetic Variants**

| **Gene** | **Mutation type** | **Variant Type** | **Inheritance** | **Ref Sequence** | **Variant** |
| --- | --- | --- | --- | --- | --- |
| ANKRD11 | Frameshift | SNV | De novo | NM_013275 | c.1893K>NfsX16 |
| ANKRD11 | Stop gained | SNV | De novo | NM_001256182 | c.1801R>X |
| ANKRD11 | Missense | SNV | Unknown | NM_001256182.1 | c.5366R>H |
| ASXL3 | Stop gained | SNV | De novo | NM_030632 | c.1783Q>X |
| BBS10 | Frameshift | SNV | Unknown | NM_024685 | c.271C>LfsX5 |
| CNOT3 | Missense | SNV | De novo | NM_014516 | c.58E>Q |
| CREBBP | Stop gained | SNV | Unknown | NM_004380 | c.1270R>X |
| CTNNB1 | Frameshift | SNV | De novo | NM_001904 | c.802_811G>WfsX5 |
| DDX3X | Splice site | SNV | De novo | NM_001356 | - |
| DDX3X | Missense | SNV | De novo | NM_001356 | c.641I>T |
| DONSON | Stop gained | SNV | Unknown | NM_017613 | c.877R>X |
| EP300 | Stop gained | SNV | De novo | NM_001429 | c.4168Q>X |
| FMR1 | Expansion | SNV | Maternal | NM_002024 | - |
| GRIN2B | Missense | SNV | De novo | NM_000834 | c.2459G>A |
| GRIN2B | Missense | SNV | De novo | NM_000834 | c.2065G>S |
| KCNQ2 | Missense | SNV | De novo | NM_172107 | c.431R>Q |
| KDM6A | Stop gained | SNV | De novo | NM_021140 | c.3200S>X |
| NF1 | - | SNV | Unknown | - | - |
| NSD1 | Frameshift | SNV | Unknown | NM_022455 | c.5279_5282V>GfsX2 |
| NSD1 | Frameshift | SNV | Unknown | NM_022455 | c.6290K>KfsX14 |
| PPM1D | Frameshift | SNV | Unknown | NM_003620.3 | c.1259S>VfsX13 |
| PPM1D | Frameshift | SNV | De novo | NM_003620 | c.1270E>GfsX10 |
| SPRED1 | Frameshift | SNV | Unknown | NM_152594 | c.395N>MfsX20 |
| TRIO | Frameshift | SNV | De novo | NM_007118 | c.863S>TfsX44 |
| UBE3A | Frameshift | SNV | Unknown | NM_130838 | c.2056R>KfsX10 |
| WAC | Stop gained | SNV | De novo | NM_100486 | c.1555R>X |
